# Supplementary material for: Putting ICU triage guidelines into practice: A simulation study using observations and interviews
Source: PLoS One. 2023 Aug 24;18(8):e0286978. doi: 10.1371/journal.pone.0286978 (PMC10449194; doi:10.1371/journal.pone.0286978)
Supplement: Attachment — Submitted filename: Response to reviewers.docx [file pone.0286978.s001.docx]

We thank the editor and the reviewers for their helpful comments. Below, a point by point response is provided.

**1. Please ensure that your manuscript meets PLOS ONE's style requirements, including those for file naming. The PLOS ONE style templates can be found at** <https://journals.plos.org/plosone/s/file?id=wjVg/PLOSOne_formatting_sample_main_body.pdf> and <https://journals.plos.org/plosone/s/file?id=ba62/PLOSOne_formatting_sample_title_authors_affiliations.pdf>
>> We have made the required adjustments. It should be noted that we have four levels of headings, but we saw no way to reduce this to three levels without making our (rather complicated) results more confusing. We have for now used italics for this fourth level (but can adjust this if needed). We have also used italics for the quotes in the text, so that they are not confused with the main text; as well as for certain phrases that correspond with the main topics that we found to provide more structure in the text. These can be removed if this is desired.

We also noted that the numbering for the tables and footnotes in the tables was not always correct. This was also adjusted.

**2. Please provide additional details regarding participant consent. In the ethics statement in the Methods and online submission information, please ensure that you have specified what type you obtained (for instance, written or verbal, and if verbal, how it was documented and witnessed). If your study included minors, state whether you obtained consent from parents or guardians. If the need for consent was waived by the ethics committee, please include this information.**
>> We have added the information that the participants signed written informed consent. The study did not include minors.

**3. Please review your reference list to ensure that it is complete and correct. If you have cited papers that have been retracted, please include the rationale for doing so in the manuscript text, or remove these references and replace them with relevant current references. Any changes to the reference list should be mentioned in the rebuttal letter that accompanies your revised manuscript. If you need to cite a retracted article, indicate the article’s retracted status in the References list and also include a citation and full reference for the retraction notice.**
>> The reference list was checked and was found to be complete and without retracted articles. We made some adjustments to the layout of some of the references and added hyperlinks to (non-article) documents such as guidelines.

**Reviewer #1: The authors have addressed a very important topic in their research. The article is written in an eye catching manner and are the methodological details have been provided. The results are presented appropriately.
Reviewer #2: The topic is very interesting & practical testing of triage guidelines in a scientific study has so far not taken place. The study findings would be helpful for hospitals adapting national guidelines.**
>> We thank the reviewers for their appreciative comments.

**I have few comments on the study:** **Well-developed methodology is there but results are bit confusing, and the numbering mentioned in table 5 are not understandable. More brief points for this should be mentioned.**
>> We agree that Table 5 is a bit of a puzzle. We only noted down the codes of the cases (rather than a description) because otherwise the table becomes very cluttered. This means the codes have to be explained outside the table. Rather than using footnotes to repeat the information in Table 3 (in which the codes are explained), we have a footnote referring to Table 3. We still believe this is the most elegant way to do it, as the footnotes section is already very long for this table. However, to better aid readers in understanding Table 5 we have added the following:

- When referring to Table 5 in the text we directly encourage readers to use Table 3 to understand the codes. P11: *Prioritization of patients was similar among the teams, though not exactly the same (Table 5; see Table 3 for case codes).*
- We have redesigned Table 3 in order to have the codes of the cases stand out more (P9). This should make it easier to look up which case each codes refers to. Additionally, we have made explicit in the title of Table 3 that this table explains the coding system used in the article.

We hope that these changes will make Table 5 easier to understand.

Additionally, while doing this, we realized that one footnote/explanation was missing from the table. We added this footnote and adjusted the letters of the other footnotes accordingly (the footnotes are also changed from numbers to letters, as per the journal’s style requirements).

**Try to elaborate introduction and discussion part as they are not properly depicting the main theme and results of your done work.**
>> We agree that the introduction section should be more elaborate. We have added a paragraph with general information regarding ICU care and COVID-19 patients in the ICU, which we feel will help readers understand the context of our research better (page 1).

Regarding the discussion section (currently 1500+ words): after discussion in the research team we remain unsure how we could better depict the main theme and results of our work, as per the reviewer’s request. Currently the discussion section covers our most important findings, compared to international guidelines and literature, and with their practical implications (i.e. need for operationalized guidelines; need for more training of triage teams; need for psychological support for triage teams); as well as a comparison with literature on prioritization in healthcare in general.

Unfortunately, we have therefore not been able to add new information to the discussion section based on the reviewer comment. We hope the reviewer will reconsider this aspect of their comment or otherwise share which topics they feel are missing from the discussion section.
